# Validation of the PREM-MSZ resulting in the PREM-Q8: a generic patient-reported experience measure for outpatient care

**DOI:** 10.1136/bmjoq-2026-004294

**Published:** 2026-10-21

**Authors:** Marloes Hendrina Paulina ter Stege, Kim Phi Luong, Harm Pieter Slijper, Ruud W Selles

**Affiliations:** 1 Xpert Clinic, Eindhoven, The Netherlands; 2 Plastic, Reconstructive and Hand Surgery, Erasmus MC University Medical Center Rotterdam, Rotterdam, The Netherlands; 3 Plastic and Reconstructive Surgery, Radboud University Medical Center, Nijmegen, The Netherlands; 4 Rehabilitation Medicine, Erasmus MC University Medical Center Rotterdam, Rotterdam, The Netherlands

**Keywords:** Patient-centred care, Quality measurement, Patient satisfaction

## Abstract

**Introduction:**

Patient experience is a key measure of healthcare quality, but many widely used Patient-Reported Experience Measures (PREMs) lack formal validation. The PREM-MSZ (‘Medical Specialist Care’) is a widely used, Dutch, policy-embedded generic PREM for hospital and outpatient care that has not yet been formally validated or translated. This study aimed to develop Dutch and English-translated brief and generic PREM based on the PREM-MSZ and evaluate its content and construct validity in outpatient care.

**Methods:**

Items were selected from the PREM-MSZ based on predefined criteria for generalisability, relevance, actionability and capturing experience. Cognitive interviews were used to assess and improve clarity, relevance and comprehensiveness. Construct validity was evaluated by analysing floor and ceiling effects, non-relevance rates and known-group validity using the Mann-Whitney U and Cliff’s delta. To test the known-groups hypothesis, we compared patients classified as promoters or detractors using the Net Promoter Score.

**Results:**

We selected eight questions based on predefined criteria and evaluated them through cognitive interviews with eight participants, confirming strong content validity. Psychometric item analyses were conducted on data from 31 120 patients across more than 25 outpatient locations and multiple medical specialties. No floor effects were found. Ceiling effects were observed mainly among promoters; among detractors, no ceiling effects were found. Known-groups analysis showed that promoters scored significantly higher than detractors on all items with large effect sizes (Cliff’s Delta>=0.75), indicating the instrument’s ability to capture meaningful differences in patient experience. Based on these findings, six closed-ended questions were retained to form the final PREM-Q8, and two open-ended questions were added to improve actionability.

**Conclusions:**

The PREM-Q8 provides initial evidence of content and construct validity and offers a brief, generic instrument for measuring patient experience in outpatient care, building on the widely used PREM-MSZ.

**WHAT IS ALREADY KNOWN ON THIS TOPIC**

- Patient experience is a key dimension of healthcare quality, yet widely used generic instruments like the Dutch Patient-Reported Experience Measure (PREM) MSZ lack formal peer-reviewed validation despite its policy-embedded and broad implementation.

**WHAT THIS STUDY ADDS**

- This study introduces the PREM-Q8, a validated, brief and generic instrument for measuring patient experience in outpatient care that balances psychometric robustness with patient usability and supports feedback-driven quality improvement.

**HOW THIS STUDY MIGHT AFFECT RESEARCH, PRACTICE OR POLICY**

- The PREM-Q8 offers a practical tool for evaluating patient experience across diverse outpatient settings.

## Introduction

 Patient experience is increasingly recognised as a key measure of healthcare quality and a driver of patient-centred care. Insight into patient experience can provide actionable insights into what matters most to patients and supports continuous quality improvement.^1–5^ The global shift towards value-based healthcare has led to a growing emphasis on consumer assessment of care quality,^6^ with patient experience being used as a key measure of value-based performance for nearly a decade.^7 8^ As a result, most health institutions have emphasised improving patient experience as a central aspect of their care delivery.^9^ In addition to informing quality improvement efforts, boosting patient experiences is also relevant because it has been associated with various meaningful consequences. For instance, more positive patient experiences have been associated with increased patient loyalty and trust,^10 11^ as well as better clinical outcomes such as pain relief and functional improvement.^12–15^ This highlights the strategic and clinical relevance of measuring patient experience in routine care.

Patient experience has been defined as ‘the sum of all interactions, shaped by an organization’s culture, that influence patient perceptions across the continuum of care’.^16^ These experiences can be systematically measured using Patient-Reported Experience Measures (PREMs), which aim to identify strengths and weaknesses in care delivery and support targeted quality improvements.^4 17^ However, psychometric evaluation of PREMs is often incomplete or lacking. A systematic review by Bull *et al*^4^ found that key aspects of measurement validity and reliability were frequently unreported or not undertaken. For example, over 60% of the PREMs questionnaires have unknown content validity and hypothesis testing.^4^ Despite the growing international use of PREMs, many routinely used instruments still lack comprehensive psychometric evaluation, particularly with respect to content and construct validity. Therefore, further validation and refinement of existing PREMs remain an important research priority.

PREMs come in various forms, including disease-specific, discipline-specific, healthcare pathway-specific and generic instruments, depending on the context in which they are intended for use. Generic PREMs assess general aspects of patient-centredness and can be used across various healthcare settings and disciplines.^18^

Several generic PREMs have been developed internationally to measure patient experience across healthcare settings. In the Netherlands, prominent examples include the Patient Experience Monitor, a Dutch adaptation of the Picker questionnaire,^19^ and the PREM-MSZ (‘Medical Specialist Care’). The present study focused on the PREM-MSZ because it is routinely implemented in Dutch specialist outpatient care and used for quality monitoring and public reporting through ZorgkaartNederland.^20 21^ The PREM-MSZ was developed between 2015 and 2017 as a concise alternative to the longer Consumer Quality Index (CQI) questionnaires. It was developed to reduce the measurement burden and enhance applicability across hospital and specialist outpatient care. Items were selected based on statistical criteria, such as non-response rates and discriminative capacity, using data from the CQI inpatient and outpatient surveys and the Ratings & Review instrument.^22^ Following this, qualitative refinement involving patient focus groups and cognitive interviews was conducted to support content validity and interpretability.^23 24^ In its latest version (V.3.0), the response scale was modified from a 5-point Likert scale to a 1–10 numeric rating scale to align with broader national reporting standards.

Despite its rigorous development process and potential to serve as a concise and generally applicable PREM in hospital and outpatient clinic settings, the latest version of the PREM-MSZ (V.3.0) has not been formally validated and is currently only available in Dutch. In this study, we build on the widely implemented PREM-MSZ by evaluating its relevance, comprehensibility, completeness and response options. Based on these insights, we refined the existing item set and evaluated its psychometric properties. To support future cross-national use, a standardised English translation was also created.

## Method

The development and validation of the PREM involved several steps: item selection, assessing content validity through cognitive interviews and evaluating psychometric properties. Reporting followed the COnsensus-based Standards for selecting health Measurement Instruments (COSMIN) Reporting Guideline 2.0; checklists are provided in online supplemental appendices F and G.

### Patient and public involvement

Patients were involved in the research by participating in cognitive interviews and completing questionnaires to evaluate the relevance, comprehensibility and completeness of the instrument. The study built upon the existing PREM-MSZ, which was originally developed with patient involvement. However, patients or the public were not involved in the design, conduct, reporting or dissemination plans of the present study.

### Item selection

To select items, we applied the earlier-mentioned definitions of patient experience as ‘the sum of all interactions, shaped by an organization’s culture, that influence patient perceptions across the continuum of care’.^16^ This concept emphasises objective aspects of care, focusing on ‘what’ happened during an episode of care and ‘how’ it happened from the patient’s perspective. In contrast, patient satisfaction captures more subjective elements, such as personal expectations and opinions about the care received.^3 4^ The resulting instrument is intended for use in hospital and outpatient clinic settings where patients typically interact directly with healthcare professionals and undergo a defined treatment process.

Based on this conceptual distinction, two authors independently selected from the PREM-MSZ V.3.0 items that (1) measured the construct of patient experience, (2) addressed patients’ interactions with the healthcare system, (3) provided information that could lead to actionable insights for improving care and (4) were broadly relevant to patients receiving care in hospitals and outpatient clinics, regardless of diagnosis or treatment type (eg, excluding items specific to medication use or other treatment-specific information). No disagreements occurred during the item selection process. Items were retained only if they met all four predefined inclusion criteria. Items that did not meet one or more criteria were either excluded or, where appropriate, modified.

### Content validity

After item selection, we conducted cognitive interviews until saturation was reached, meaning no new issues requiring modifications to the questionnaire were identified.^25^ Participants were recruited from two specialty clinics within the same outpatient healthcare organisation. Eligible participants were at least 18 years old and proficient in Dutch and had received treatment at a clinic within the previous 3 months. One of the specialty clinics offers specialised, insured care for hand and wrist surgery and therapy, proctology, eye care and orthopaedic surgery. The other clinic specialises in uninsured care for plastic surgery and cosmetic treatments. To minimise bias, participants were recruited from these two distinct outpatient clinics with different specialties, representing a diverse patient population across more than 25 locations in the Netherlands. To reduce potential selection bias, a random sample of eligible patients was invited to participate.

To maximise insight from the validation, we used the Think-Aloud Protocol and Verbal probing method for complementary insights.^26–28^ Think-Aloud Protocol required participants to verbalise their thoughts while answering questions without the interviewer’s direction.^29 30^ Verbal probing included probing questions about relevance, comprehensibility, recall period and comprehensiveness, following COSMIN guidelines.^31^ Examples include the following: ‘Is the item clearly stated?’, ‘How relevant is this item to your experience?’ and ‘How did you come up with your answer? Why didn’t you score higher or lower?’.

After conducting the first three interviews, we performed an interim analysis to assess whether modifications to the questionnaire or response scales were warranted. This iterative approach allowed us to refine the instrument based on initial participant feedback before proceeding with subsequent interviews.

To ensure consistency and comparability, the interviews were conducted via video and audio recorded by the author, who is most experienced in patient contact and familiar with the Cognitive Interviewing Training Guide.^32^ Another author transcribed the recordings verbatim and anonymised the transcripts. The data from all interviews were analysed and categorised independently by both authors using the classification scheme of Willis and Lessler^33^ as recommended by Buers *et al*^34^ (see online supplemental appendix A). A third author made the final decision with divergent interpretations. The final PREM-Q8 English and Dutch versions of the questionnaire are provided in online supplemental appendices B and C.

All participants received an informative letter and provided written consent. Data were stored anonymously on a secure server.

### Data acquisition and analysis

After item selection, we evaluated the measurement properties of the refined PREM. Patients (aged 18+) receiving surgical or conservative care at our clinics between January 2022 and March 2025 were offered the PREM questionnaire 3 months after treatment as part of our routine outcome measurement.^35^ Psychometric analyses were based on all available complete questionnaires collected during the study period; therefore, no formal sample size calculation was performed. Patients reported their experiences throughout the entire treatment episode. The six closed-ended items are rated on a 10-point numeric rating scale (1 = ‘No, not at all’; 10 = ‘Yes, completely’), with higher scores indicating more positive experiences. The survey also included the Net Promoter Score (NPS) question to enable known-group comparisons. Non-responders received up to two reminder emails. Only complete responses were included in the analysis.

Data collection and management were performed using GemsTracker electronic data capture tools. GemsTracker (GEneric Medical Survey Tracker) is a secure web-based application for distributing questionnaires during medical research and quality studies. All data were processed using R V.4.4.2 (R Foundation for Statistical Computing, Vienna, Austria).

### Descriptive statistics

To provide an overview of the study sample, the characteristics of participants (eg, sex, age, health status, care domain) were summarised (table 1).

**Table 1:** Characteristics of participants (N=31 120)

Description	N	%
Sex		
Female	19 719	63
Male	11 221	36
Unknown	180	1
Age		
18–30	2101	7
31–45	4106	13
46–60	9878	32
61–75	12 151	39
>75	2884	9
Health status		
Bad	258	1
Poor	3240	10
Good	14 929	48
Very good	9257	30
Excellent	3436	11
Treatment domain		
Plastic surgery	3732	12
Cosmetic dermatology	789	3
Hand and wrist	18 632	60
Eye surgery	1683	5
Orthopaedics	4410	14
Proctology	1874	6
Satisfaction with (preliminary) treatment result		
Low (1–4)	2217	7
Medium (5–6)	4638	15
High (7–8)	11 654	37
Very high (9–10)	12 611	41
NPS classification		
Detractors (1–6)	2387	8
Neutrals (7–8)	9517	31
Promoters (9–10)	19 216	62

NPS, Net Promoter Score.

### Psychometric properties

To evaluate the psychometric properties of the PREM, we focused on construct validity in accordance with the COSMIN standards for psychometric evaluation. The PREM uses a formative measurement model, meaning each item represents a unique aspect of the patient experience. Therefore, no overall summary score was calculated, and analyses assuming reflective models, such as factor analysis, item response theory and internal consistency measures, are inappropriate.^36 37^

We first assessed ceiling and floor effects and non-relevant rates. Ceiling and floor effects occur when a large proportion of participants (15% or more) selected the highest (10) or lowest (1) possible score.^38^ Non-relevant responses were calculated as the percentage of ‘not applicable’ answers per item. Items with a non-relevant rate over 5% should be reviewed for relevance, as they may target a specific population.

Next, we evaluated known-group validity to determine if the PREM can differentiate between groups with expected differences in experiences.^39^ The NPS is based on the Friends and Family Test question: ‘Would you recommend this clinic to friends and family if they needed similar care?’. The NPS was selected because willingness to recommend a clinic has been shown to be strongly associated with their experience with care.^40^ Participants scoring 9–10 were classified as promoters, 7–8 as neutrals and 1–6 as detractors.^41 42^ We hypothesised that promoters would report more positive experiences than detractors. To test this, mean item scores were compared between the two groups, excluding neutrals to focus on the extremes.

Given the unequal group sizes and non-normal distribution, the Mann-Whitney U test was used with a significance threshold of p<0.05. Effect sizes were calculated using Cliff’s delta, with thresholds of 0.11–0.27 (small), 0.28–0.42 (medium) and ≥0.43 (large).^43^

### Patient-Reported Experience Measure translation

To facilitate future international research, the final Dutch PREM-Q8 was translated into English using a standardised forward-backward procedure based on key principles outlined by Wild *et al*.^44^ This translation was intended to produce a conceptually equivalent English version and was not designed as a formal cross-cultural adaptation or validation according to international translation guidelines. First, a native English speaker fluent in Dutch translated the original Dutch version into English. Next, a second translator, native in Dutch, fluent in English and unfamiliar with the PREM, back-translated the English version into Dutch. The original Dutch version and the back-translation were then compared to identify any discrepancies, changes in meaning or subtle shifts in interpretation. Based on this comparison, minor adjustments were made to the English translation to ensure conceptual equivalence and clarity. The final English version of the PREM is provided in online supplemental appendix B.

## Results

### Item selection

Six of the nine original items met all four predefined criteria (table 2). The treatment effect (item 7) and recommendation items (item 8) were excluded because they do not directly measure the patient experience and offer a limited basis for actionable care improvements. Furthermore, the open-ended item 9 was split into two separate items to enhance actionability: one for suggestions for improvement and one for compliments. The remaining items formed a preliminary set of eight items (see online supplemental appendix B).

**Table 2:** Evaluation of original PREM items based on inclusion criteria

Original item	Measures patient experience	Focuses on interaction with health system	Leads to actionable insights	Relevant to the general population	Inclusion decision
1. Did the healthcare provider(s) listen to you carefully?	Y	Y	Y	Y	Retained
2. Did you understand the explanation from the healthcare provider(s)?	Y	Y	Y	Y	Retained
3. Did you have confidence in the expertise of the healthcare provider(s)?	Y	Y	Y	Y	Retained
4. Were the advantages and disadvantages of the treatment or surgery explained to you?	Y	Y	Y	Y	Retained
5. Did you and the healthcare provider(s) decide together on your care or treatment?	Y	Y	Y	Y	Retained
6. Was there a good collaboration between the healthcare providers in the clinic?	Y	Y	Y	Y	Retained
7. How do you rate the (preliminary) effect of your treatment?	N	Y	N	Y	Excluded—does not measure patient experience but rather treatment outcome; also does not yield actionable insights for experience improvement.
8. Would you recommend this healthcare provider to other people with the same condition or health issue?	N	Y	N	Y	Excluded—measures overall satisfaction or loyalty rather than experiences; does not offer actionable insights for quality improvement.
9. What are you very satisfied with in terms of healthcare provision? What do you think could be improved in the provision of care?	Y	Y	N	Y	Modified: We generated two open-ended questions to improve actionability (see online supplemental appendix B)

Item 7 focuses on outcome rather than care experience.

Item 8 was excluded as it reflects satisfaction rather than experience and provides limited actionable insight.

Item 9 was split into two questions to improve actionability: one asking for suggestions for improvement and one for compliments.

N, criterion not met; PREM, Patient-Reported Experience Measure; Y, criterion met.

### Content validity

We conducted eight cognitive interviews with all female participants (mean age 39 years; range 21–68 years). Participants represented different treatment specialties, including hand and wrist surgery, orthopaedics, proctology and plastic surgery, with several participants having experience across multiple specialties. We attempted to recruit approximately 25 eligible male patients. However, none completed the interviews because they could not be reached, declined participation when contacted or withdrew before the scheduled interview.

17 issues were identified across the interviews (see table 3). After the first three interviews, it became apparent that patients had difficulty differentiating among the highest response options (8–10) on the original 1–10 scale. Based on these findings, participants in the subsequent interviews evaluated both the original response scale and an alternative response scale, in which 6 indicated ‘Yes’ and 10 represented ‘Strongly agree’. Most participants favoured the original 1–10 scale, which all participants could use without difficulty.

**Table 3:** Results of the cognitive interviews

Theme	Number of problems	Key findings
Instructions	1	The introduction text mentions the abbreviation PREM-MSZ, but it is unclear to one patient what it means.
Clarity	1	One participant was uncertain whether ‘collaboration between healthcare providers’ referred to the full care pathway or only the treatment itself.
Assumptions	2	Participants were unsure all other contacts with the clinic (besides the treatment) or aftercare provided at a different location were included in the items’ scope.
Knowledge/memory	1	One participant indicated that they had not consciously reflected on their trust in the provider’s expertise at the moment of care. The response was based on general assumptions or prior expectations, rather than a recollection of a specific interaction.
Sensitivity/bias	1	One participant commented that the open-ended question about giving a compliment might feel inappropriate for patients who had a negative experience.
Response categories	7	Participants found it difficult to distinguish between high scores (eg, 8–10), which led to hesitation and longer response times. Despite this, they gave different scores, suggesting some response variability. Several participants suggested that a more concise scale (eg, 1–5) or labelled options such as ‘Never’, ‘Sometimes’, ‘Often’ and ‘Always’ would be more intuitive.
Other problems	4	Some participants found it difficult to answer the item about collaboration between healthcare providers due to a lack of visibility into all aspects of the collaboration. They responded based on what they personally observed. The shared decision-making item was perceived as irrelevant by some participants, as some patients had no opportunity to participate in the decision-making process because it was already clear what the treatment should be.

Shown are the problems identified during cognitive interviews (n=8), grouped by theme.

The number of problems reflects the number of distinct comments within each theme.

Key findings describe representative examples or recurring issues raised by participants.

PREM, Patient-Reported Experience Measure.

Verbal probing confirmed that the items were considered relevant, clear and comprehensive. Participants reported that the questionnaire adequately captured their healthcare experience and did not identify any key topics that were missing. When explaining their answers, participants could clearly articulate why they gave the scores they did, including why they did not choose higher or lower ratings.

Based on these insights, we decided not to modify the questionnaire items or response scale (see online supplemental appendix B). The finalised version, consisting of six closed-ended items and two open-ended items, is hereafter referred to as the PREM-Q8.

#### Data analysis of psychometric properties

A total of 99 539 patients were invited to complete the PREM questionnaire. Of these, 77 124 provided informed consent, and 31 120 completed the questionnaire and were included in the psychometric analyses after exclusion of incomplete questionnaires (online supplemental appendix D). Compared with non-responders, responders were slightly older (mean age 57.7 vs 53.6 years; standardised mean difference (SMD) = 0.25), whereas the sex distribution was comparable (63.4% vs 63.3% female; SMD=0.03) (online supplemental appendix E). Table 1 presents an overview of participant characteristics, care context and patients rating of the (preliminary) treatment result.

### Construct validity—item performance

No floor effects were found. We found ceiling effects in all items in the total and equal-sized groups (see table 4); however, when selecting only the detractors as classified by the NPS, none of the items showed a ceiling effect.

**Table 4:** Results of item performance measures

Items	Ceiling effect* (%)			Floor effect* (%)	**Non-relevant rate****†** **(%)**
	Total population (n=31 120)	Equal-sized groups‡ (n=7161)	Detractor§ (n=2387)		
Did the healthcare provider(s) listen to you carefully?	53.6	36.7	12.5	0.3	1.1
Did you understand the explanation from the healthcare provider(s)?	53.1	36.4	13.6	0.2	0.9
Did you have confidence in the expertise of the healthcare provider(s)?	55.5	37.8	12.1	0.3	0.7
Were the advantages and disadvantages of the treatment or surgery explained to you?	47.0	30.9	9.7	1.6	7.9
Did you and the healthcare provider(s) decide together on your care or treatment?	48.7	32.5	11.6	1.6	5.0
Was there a good collaboration between the healthcare providers in the clinic?	52.4	35.3	11.5	0.6	7.0

A high ceiling effect may indicate limited ability to discriminate between high-performing providers*.*

* Ceiling and floor effects reflect the percentage of respondents selecting the most (ceiling) or least (floor) positive response option*.*

† The ‘non-relevant rate’ shows how often respondents indicated the item did not apply to them.

‡ ‘Equal-sized groups’ refers to groups created by randomly sampling an equal number of respondents from each NPS group, using the smallest group as the reference.

§ The ‘detractor’ group includes respondents who gave a score between 1 and 6.

NPS, Net Promoter Score.

The non-relevant rate, defined as the proportion of participants who answered ‘not applicable’ to a given item, exceeded 5% for two of the six items: item 4 (advantages and disadvantages of the treatment or surgery) and item 6 (collaboration between healthcare providers). This suggests that these items may not be relevant to all patients and should be carefully reviewed to assess whether they are appropriate for inclusion across the entire target population.

### Construct validity—known-groups validity

Promoters on the NPS scored significantly higher on all items than detractors (see table 5), suggesting that the PREM-Q8 can distinguish between patients with different levels of patient experience. Across all items, the SD was substantially higher among detractors than promoters, indicating more variability and heterogeneity in their experiences. The differences between the two groups were statistically significant (all p<0.001), and the Cliff’s delta for all items was consistently 0.75 or above, indicating large effect sizes and demonstrating that the PREM effectively discriminates between patients with different experiences.

**Table 5:** Known-groups validity

Items	**Promoters (mean±SD)**	**Detractors** **(mean±SD)**	**P** value	Cliff**’**s **d**elta
Did the healthcare provider(s) listen to you carefully?	9.6±0.8	6.8±2.3	<0.001	−0.78
Did you understand the explanation from the healthcare provider(s)?	9.6±0.8	7.1±2.1	<0.001	−0.76
Did you have confidence in the expertise of the healthcare provider(s)?	9.7±0.7	7.0±2.1	<0.001	−0.80
Were the advantages and disadvantages of the treatment or surgery explained to you?	9.3±1.3	6.0±2.6	<0.001	−0.77
Did you and the healthcare provider(s) decide together on your care or treatment?	9.4±1.2	6.2±2.7	<0.001	−0.75
Was there a good collaboration between the healthcare providers in the clinic?	9.6±0.8	6.5±2.5	<0.001	−0.79

Known-groups validity was assessed by comparing mean item scores between promoters (respondents who scored nine or 10 on the NPS recommendation question) and detractors (score 1–6).

All comparisons were statistically significant at p<0.001, supporting the instrument’s discriminative validity.

Cliff’s delta is a non-parametric effect size measure; negative values indicate lower scores among detractors

NPS, Net Promoter Score.

## Discussion

This study describes the development and validation of a commonly used PREM for outpatient care. We selected items based on predefined criteria and conducted cognitive interviews, resulting in an eight-item version with strong content validity. Participants found the items clear, relevant and comprehensive. Psychometric analyses showed no floor effects. Ceiling effects were largely confined to promoters, while detractors showed none. The PREM-Q8 demonstrated initial construct validity through a known-groups comparison, with large effect sizes in distinguishing promoters from detractors, although discrimination at the upper end remained limited.

Our findings confirm that the included items are relevant and easily understood by patients. While some items prompted clarification requests, such as whether contacts outside the treatment itself were intended to be included in the response, no fundamental changes were deemed necessary, as all patients could easily complete the questionnaire. Although participants reported no missing essential domains, the group was relatively small. Most participants favoured the 1–10 response scale over the alternative unbalanced version despite its tendency to increase ceiling effects. This preference highlights the challenge of balancing psychometric performance with ease of use.

While content validity was strong, two items (‘Cooperation of care provider(s)’ and ‘Pros and cons are told’) exceeded the 5% threshold for perceived irrelevance, possibly due to limited contact with healthcare professionals. Nevertheless, both items were judged relevant in the cognitive interviews and have conceptual importance, such as good team collaboration, which is essential for safe care.^45^

Ceiling effects were observed across all items, consistent with previous studies of patient experience measures.^46–48^ While no floor effects were present, ceiling effects reduce measurement sensitivity and hinder the ability to detect change or evaluate quality improvement initiatives. In our cognitive interviews, many participants stated that their care ‘couldn’t have been better’, reporting no unmet expectations. Regardless of whether ceiling effects indicate genuinely high performance or scale limitations, restricted variability reduces the capacity of quantitative scores for informing targeted improvement. High performance may leave limited room for further optimisation. In such cases, open-ended responses complement quantitative scores by capturing specific strengths and suggestions for improvement that structured items may overlook, despite not resolving the psychometric limitations associated with ceiling effects. Our data show that equal weighting of promoter and detractor groups (ie, correcting for sample imbalance) reduced ceiling effects by up to 18 percentage points on some items. This suggests that ceiling effects reflect not only instrument design but also the sampling distribution. Although ceiling effects limit discrimination among patients reporting very positive experiences, the instrument remained capable of distinguishing between patients with substantially different experience levels and therefore appears suitable for its intended purpose of supporting quality improvement.

Although unbalanced response scales may reduce ceiling effects,^49^ participants preferred a standard 1–10 scale and considered the alternative less intuitive. Previous studies have likewise shown only limited success in reducing ceiling effects through response-scale modifications.^46 50–52^ Despite some difficulty distinguishing adjacent high scores (eg, 8 vs 9), participants consistently justified their ratings based on specific aspects of care, suggesting that these scores still reflect meaningful experiential variation.

Although open-ended questions were not explicitly intended to mitigate ceiling effects, they provide a valuable complement by capturing issues not reflected in quantitative scores.^53^ Detractor comments are often particularly detailed and rich in improvement suggestions.^54 55^ We therefore recommend using qualitative comments alongside quantitative PREM scores to guide quality improvement. Future research should further explore the thematic content of detractor comments, assess item-level discrimination and evaluate hybrid approaches such as stratified or adaptive PREM administration.

The instrument showed evidence of known-groups validity by effectively differentiating between NPS-defined promoters and detractors. These findings provide initial support for the construct validity of the PREM-Q8. The large effect sizes suggest that the questionnaire is sensitive to meaningful differences in patient experience relevant to quality improvement. However, construct validity was evaluated using a single known-group comparison based on the NPS. Future studies should examine additional known groups and complementary approaches to further strengthen the evidence for construct validity. Whereas this study was conducted within a single outpatient healthcare organisation, data were collected across more than 25 outpatient locations, involving over 100 physicians across various specialties, including ophthalmology, orthopaedics, proctology, plastic surgery, hand and wrist surgery, and cosmetic dermatology. This multisite approach strengthens the potential generalisability of the instrument beyond individual practices. In addition, responders were only slightly older than non-responders, while the sex distribution was comparable, suggesting limited responder bias based on the available demographic characteristics. Furthermore, the instrument is based on the PREM-MSZ, which builds upon the concepts and domains initially established by the CQI developed for Dutch hospital care.^24 56 57^ Still, further research is needed to confirm its usefulness in other healthcare environments.

This study is strengthened by its contribution to the growing body of research on patient experience, as it provides a validated instrument for the Dutch outpatient clinic setting. We validated the widely used, policy-embedded PREM-MSZ through a combination of cognitive interviews and psychometric testing in a large and diverse sample. The resulting PREM-Q8 is both scientifically robust and practically relevant.

While promising, this study also has several limitations. First, test-retest reliability, responsiveness and internal consistency were not evaluated. The latter was not assessed because the PREM-Q8 was developed to capture multiple distinct aspects of patient experience rather than a single homogeneous construct. Second, psychometric analyses were conducted within a single outpatient clinic, limiting generalisability. Third, cognitive interviews included only a small group of female participants. Although saturation was reached, the absence of male perspectives may have limited the identification of gender-specific issues, as studies have reported gender differences in patient experience and care priorities,^58–60^ which may have influenced the comprehensiveness of the final item set. We also did not assess convergent validity with other established PREMs or additional known-group comparisons. Fourth, the PREM-Q8 was not designed to incorporate advanced methodological strategies to reduce ceiling effects. Finally, the English version requires formal cross-cultural validation before use in international settings.

Future studies should address these gaps by evaluating the reliability and responsiveness, including more diverse populations and examining correlations with existing PREMs.

In conclusion, this study introduces the PREM-Q8: a validated, brief, generic instrument for measuring patient experience in outpatient care. It strikes a balance between psychometric robustness and patient usability, effectively distinguishes between patient subgroups and provides meaningful feedback by combining its quantitative output with qualitative responses.

## Supplementary material

10.1136/bmjoq-2026-004294 online supplemental file 1

## Data Availability

Data are available upon reasonable request.
