# Supplementary material for: Validation of the PREM-MSZ resulting in the PREM-Q8: a generic patient-reported experience measure for outpatient care
Source: BMJ Open Qual. 2026 Oct 21;15(4):e004294. doi: 10.1136/bmjoq-2026-004294 (PMC13691872; doi:10.1136/bmjoq-2026-004294)
Supplement: Supplementary file 1 [file bmjoq-15-4-s001.docx]

**Appendix A.**

Classification Scheme (adapted from G. B. Willis & Lessler (1999)) for identifying problems for the PREM questionnaire. This classification scheme was used to categorize common problems identified during cognitive interviews. It guided the systematic evaluation of the PREM questionnaire items to enhance clarity, relevance, and patient understanding.

| **Category** | **Explanation** |
| --- | --- |
| Instructions | Problems with introductions, instructions, or explanations |
| Clarity | Problems with the intent or the meaning of an item |
| Assumptions | Problems with assumptions or underlying logic |
| Knowledge | Likely to not known or have trouble remembering information |
| Sensitivity | Sensitive nature or wording/bias |
| Response categories | Problems with the response categories, due to the lack of an appropriate answer to the item, due to a mismatch between the question and the answer, or due to insufficient disease-specific goals |
| Other problems | Problems with layout or item ordering |

**Appendix B**

Final English version of the eight selected PREM items and their scoring after item selection, cognitive testing (content validity), and translation from Dutch.

**Appendix C**

Dutch version PREM-Q8

**Appendix D**

Flow diagram of patient inclusion in the psychometric analyses.

**Appendix E**

Comparison of baseline characteristics between responders and non-responders.

Data are represented as mean (SD) or n (%). Standardized mean differences (SMDs) were calculated to quantify differences between responders and non-responders; values <0.10 indicate negligible differences, 0.10-0.20 small differencecs, and >0.20 potentially meaningful differences.

| **Characteristic** | **Non-responders (n = 46,004)** | **Responders (n = 31,120)** | **Standardized mean difference** |
| --- | --- | --- | --- |
| Age, years, mean (SD) | 53.57 (17.03) | 57.65 (14.97) | 0.25 |
| **Sex**, n (%) |  |  | 0.03 |
| Female | 29,131 (63.3) | 19,719 (63.4) |  |
| Male | 16,499 (35.9) | 11,221 (36.1) |  |
| Unknown | 374 (0.8) | 180 (0.6) |  |

**Appendix F**

COSMIN Reporting Guideline 2.0 checklist

| **COSMIN item** | **Item description** | **Reported?** | **Manuscript location** |
| --- | --- | --- | --- |
| **T1** | Identify the report as a study of one or more measurement properties of a specific PROM to measure a specified construct in a specified population. | Yes | Title |
| **A1** | Provide the specific objective(s) of the research, specifying (1) the name (and version, if relevant), and construct(s) of the PROM, (2) the measurement properties being evaluated, and (3) relevant study characteristics. | Yes | Abstract – Introduction |
| **A2** | Specify (details of the) study design used to evaluate the measurement properties. | Yes | Abstract – Methods |
| **A3** | Specify the methods for evaluating each measurement property. | Yes | Abstract – Methods |
| **A4** | Provide the main results for all measurement properties evaluated. | Yes | Abstract – Results |
| **A5** | Provide a brief statement of the implications of the findings in the context of existing evidence on the PROM. | Yes | Abstract – Conclusions |
| **I1** | Specify the name and, if relevant, the version, and construct(s) of the PROM. | Yes | Introduction |
| **I2** | Specify the target population and context of use that the PROM was designed for. | Yes | Introduction |
| **I3** | Provide a description of the current scientific knowledge regarding the measurement properties of the PROM. Explain why the new study is necessary. Provide citations for the original development paper(s). | Yes | Introduction |
| **I4** | Provide the specific objective(s) of the research, specifying (1) the name (and version, if relevant) of the PROM, (2) the measurement properties being evaluated, and (3) relevant study sample characteristics. | Yes | Introduction |
| **GM1** | Specify (details of the) study design used to evaluate the measurement properties. | Yes | Methods |
| **GM2** | Specify how the study participants were selected. Specify the inclusion and exclusion criteria. | Yes | Methods – Content validity; Data acquisition and analysis |
| **GM3** | Provide details about the original version of the PROM as well as of the PROM version being studied, specify the conceptual framework (reflective/formative model), details on the structure (number of items and subscales), language, response scale, recall period, direction of scoring, scoring algorithm, and how the PROM was administered, including the country in which it was administered. | Yes | Introduction; Methods; Appendices B–C |
| **GM4** | Describe why and how other data were collected (e.g., comparator instruments, characteristics of comparison groups, rationale for choosing groups), including mode of administration. | Yes | Methods – Data acquisition and analysis |
| **GM5** | Provide all time points of all measurements. | Yes | Methods – Data acquisition and analysis |
| **GM6** | Provide a rationale for the sample size for all measurement property analyses (including subgroups). | Yes | Methods – Content validity; Data acquisition and analysis |
| **GM7** | Describe the statistical analyses corresponding to all objectives. Describe the criteria for good measurement properties. Name the statistical package used and the version. | Yes | Methods – Psychometric properties |
| **GM8** | Describe approaches for dealing with missing data. | Yes | Methods – Data acquisition and analysis; Results (Appendix D) |
| **GM9** | Specify analyses that were unplanned and their rationale. | N/A | — |
| **GR1** | Provide study participants' characteristics, specified per subgroup if applicable. | Yes | Results – Table 3; Appendix E |
| **GR2** | Provide the total number of participants included in the study and the sample size for each analysis. | Yes | Results; Appendix D |
| **GR3** | Provide amount of (proportion or count) and reasons for missing data for each analysis for the PROM and for any analyses of other outcome measurement instruments. | Partly | Results – Appendix D |
| **GR4** | Describe the results corresponding to all objectives (see measurement property-specific boxes). | Yes | Results |
| **DC1** | Provide the main findings and indicate whether each evaluated measurement property was sufficient or insufficient, and why. | Yes | Discussion |
| **DC2** | Discuss the practical relevance of the findings in terms of recommendations for (not) using the PROM. | Yes | Discussion |
| **DC3** | Discuss strengths and limitations of the study, including potential sources of bias that may have influenced the results. | Yes | Discussion |
| **DC4** | Discuss the generalizability of the results. | Yes | Discussion |
| **DC5** | Discuss what modifications are needed to the existing PROM. | Yes | Discussion |
| **DC6** | Describe future research questions or hypotheses generated by the findings and the research needed to address them. | Yes | Discussion |
| **DC7** | Provide the overall conclusions regarding the use of the PROM. | Yes | Discussion – Conclusion |
| **O1** | State any conflict of interest related to the PROM (e.g., involvement in PROM development, commercial funding, financial interests). | Yes | Declarations – Competing interests |

**Appendix G**

COSMIN Measurement Property Reporting Modules.

Only the COSMIN reporting modules applicable to the measurement properties evaluated in this study (content validity and hypothesis testing for construct validity) are reported.

| **COSMIN item** | **Item description** | **Reported?** | **Manuscript location** |
| --- | --- | --- | --- |
| **CV1** | Specify if and how patients and/or professionals were asked whether the instructions, each item, response options, and recall period were relevant for the construct, population, and context of use. | Yes | Methods – Content validity |
| **CV2** | Specify whether and how patients and/or professionals were asked whether all key concepts are included in the PROM. | Yes | Methods – Content validity |
| **CV3** | Specify whether and how the comprehensibility of the instructions, items, response options, and recall period was evaluated by patients and/or professionals. | Yes | Methods – Content validity |
| **CV4** | Specify whether the instructions, items, response options, and recall period were considered relevant by patients and/or professionals for the construct, population, and context of use. | Yes | Results – Content validity |
| **CV5** | Specify whether patients and/or professionals considered all key concepts to be included in the PROM. | Yes | Results – Content validity |
| **CV6** | Specify whether patients understood the instructions, items, response options, and recall period as intended and/or whether professionals considered them appropriately worded. | Yes | Results – Content validity |
| **ConV1** | State hypotheses and provide the rationale for each hypothesis. | Yes | Methods – Psychometric properties |
| **ConV2** | Specify all statistical methods used to test the hypotheses. | Yes | Methods – Psychometric properties |
| **ConV3** | Provide all results and specify whether each result was in accordance with its hypothesis. | Yes | Results – Psychometric properties |
